# Isolation and Activity Evaluation of Callus-Specific Promoters in Rice (*Oryza sativa* L.)

**DOI:** 10.3390/genes16050610

**Published:** 2025-05-21

**Authors:** Xiaojiao Ma, Chuanyin Wu

**Affiliations:** State Key Laboratory of Crop Gene Resources and Breeding, Institute of Crop Sciences, Chinese Academy of Agricultural Sciences, Beijing 100081, China; maxiaojiao85@163.com

**Keywords:** callus, specific promoter, *GUS* reporter gene, genetic transformation, gene editing, rice

## Abstract

**Background/Objectives:** In crop genetic engineering, morphogenic genes have attracted increasing attention, given their ability to facilitate the transformation of a broad range of otherwise nontransformable cultivars. However, few callus-specific promoters have been identified to date that can be employed to avoid the adverse effects resulting from the ectopic expression of morphogenic genes on shoot regeneration and growth. **Methods:** A set of potential callus-specific genes were initially selected based on publicly available data. These genes were then screened using quantitative real-time polymerase chain reaction (qPCR), followed by promoter activity evaluation using a transgenic approach with the *GUS* gene serving as a reporter. **Results**: Of the 24 evaluated promoters, 12 were verified as being callus-specific using qPCR. Five genes (*Os11g0295900*, *Os10g0207500*, *Os01g0300000*, *Os02g0252200*, and *Os04g0488100*) were chosen, and their promoters were cloned. Based on GUS staining, the *pOsTDL1B* (*Os10g0207500*) promoter showed strong callus-specific expression, *pOsEDC* (*Os01g0300000*) was a medium-level callus-specific promoter, and *pOsDLN53* (*Os02g0252200*) was strictly callus-specific, although its activity was low. Quantification of GUS activity indicated that all three *pOsTDL1B:GUS* transgenic lines exhibited strong callus specificity relative to the various tissues tested. **Conclusions:** A callus-specific promoter was identified that can be used to drive the expression of morphogenic genes in crop transformation.

## 1. Introduction

Rice (*Oryza sativa* L.) is a critical food crop globally, serving as a staple food resource for approximately 3.5 billion people, which is more than half of the world’s population [1]. China and India jointly contributed ~50% to the global cultivation and consumption of rice. Overall, rice provides up to 50% of the dietary energy supply to millions of impoverished people in Asia [1], thereby making a significant contribution to global food security. Traditional breeding methods and modern cultivation have been employed to enhance its agronomic traits as well as increase its yield and quality. In China, the average rice yield has risen to approximately 6 t/ha [2]. Cooking quality (amylose content), milling quality (head rice percentage), and appearance quality (chalkiness degree) are rice grain quality components [3]. Transgenic technology is not only a key component in molecular biology research but also represents a powerful tool in rice (*O. sativa* L.) breeding [4,5]. It can be employed for the development of desirable agronomic traits, such as improved biotic/abiotic stress resistance, yield, and quality. It also offers significant production potential for dietary supplementation and food processing [6]. Blast-resistant rice was obtained by knocking out the *OsERF922* gene [7]. Low-cadmium *indica* rice was developed by knocking out the *OsNramp5* gene [8]. Simultaneously knocking out the three weight-related genes, *GW2*, *GW5*, and *TGW6*, significantly increased grain weight [9]. Editing of the *Waxy* gene could develop rice cultivars with suitable amylose content to increase the eating quality [10]. In addition, the development of golden rice provided an opportunity to minimize vitamin A deficiency in areas where rice is the staple food [11]. For animal feed, a phytase gene has been expressed in the maize endosperm to improve phosphorus availability and reduce the need for supplemental phosphorus, potentially benefiting animal nutrition and the environment [12]. Another advantage of transformation technology is that it can transfer genes controlling agronomically useful traits between species that have reproductive barriers [4].

Genome editing technologies, such as the CRISPR/Cas9 system, can efficiently and precisely create mutations in almost every gene in a genome, thus becoming increasingly popular in both basic biological research and trait development in crops. In most cases, however, high genome editing efficiency relies on efficient transformation technologies. First, the delivery efficiency of genome editing reagents to plant cells is one of the key factors in realizing high-throughput genome modification, which is largely related to the transformation method. Second, a key step in transformation is regenerating plants from cells that have been modified by genome editing. Therefore, recent rapid progress in gene editing technology development has necessitated the development of genetic transformation methods that can be used to generate large-scale high-precision genomic changes, especially in broader genetic backgrounds of various crops [13,14]. In general, transformation systems enable us to analyze gene regulatory networks, such as in transient assays, to produce plants over-expressing genes of interest, and to knock out or knock down genes for functional analysis. However, it is challenging to transform recalcitrant varieties of major crops.

Over the past few decades, considerable progress has been made in improving transformation efficiency by genotype selection, medium testing, and tissue culture condition optimization, in many major crops, including rice (*O. sativa* L.), wheat (*Triticum aestivum* L.), maize (*Zea mays* L.), and sorghum (*Sorghum bicolor* L.) [15,16,17,18,19]. It is typically performed using *Agrobacterium*-mediated transformation or particle bombardment [20]. Plants generated through the particle bombardment method often contain multiple copies of transgenes integrated at multiple genome sites, which can complicate genetic analysis in following generations and may also cause gene silencing [4,21,22]. The significant advantages of the *Agrobacterium*-mediated transformation method are its low cost and efficiency, its ability to achieve a large number of single-copy or low-copy transgene insertions, and its capacity to transfer large DNA fragments into plant genomes. Accordingly, this remains the method of choice for plant transformation [23]. The transition from the gene gun method to *Agrobacterium*-mediated genetic transformation represented both a significant technological advance and a further enrichment and refinement of the genetic transformation protocol [24].

Callus is a frequently used explant for genetic transformation. High-throughput transformation systems in major crops almost always start with callus initiation as the first step [25], followed by antibiotic selection, and, finally, the regeneration of plantlets from transformed calli. Quality callus (often defined as embryogenic callus) is the key to efficient callus transformation. However, initiation of embryogenic callus formation is challenging in most cereal crops and is highly genotype dependent [25]. In addition, embryogenic callus is also an ideal explant for quickly testing genome editing tools, owing to the highly active cells contained within and easier infection by *Agrobacterium* [20]. However, little progress has been made in embryogenic callus induction in the past few decades by conventional tissue culture methods. Calli obtained from immature embryos or those derived from mature seeds have proven to be the most suitable transformation explants in most cereal crops, including *Avena sativa* L., *Hordeum vulgare* L., *O. sativa* L., and *S. bicolor* L. Other explants have included immature inflorescence of *S. bicolor* L., leaf of *A. sativa* L. and *T. aestivum* L., and shoot apices of *O. sativa* L. [26,27,28,29,30]. Despite the tremendous and long-term efforts to improve tissue culture conditions, genotype-dependence and low transformation efficiency are considered to be technical bottlenecks in the gene-editing process [15,31]. Morphogenic transcription factor (MTF)-encoding genes represent a promising tool for use in plant transformation, facilitating the reprogramming of somatic cells to pluripotency and thereby driving embryogenesis and enhanced regeneration efficiency of transgenic plants [32]. The morphogenic genes Baby boom (*Bbm*), Wuschel-related homeobox 5 (*WOX5*), and Wuschel (*Wus*) have been recently used to improve transformation efficiency, shorten transformation time, and overcome both genotype and species dependence [33,34,35]. However, the ectopic overexpression of these genes can result in undesired pleiotropic phenotypes, such as lethal or dwarfed regenerants, and infertility of transgenic plants that happen to survive transplanting [33,36]. Therefore, the use of callus-specific promoters to drive those genes may avoid the side effects while still retaining their role in boosting the induction of embryogenic calli [32,37].

Promoters dictate the timing, location, and levels of transgene expression. In plant transformation, only a few tissue-specific promoters have been tested to drive defined expression patterns of transgenes [37]. Several strong constitutive promoters are commonly utilized in the creation of transgenic plants [38,39,40]. Using tissue-specific promoters instead of constitutive ones to drive gene expression can both allow for the accumulation of gene products within a specific timeframe and location as well as significantly reduce the metabolic burden and adverse impacts on plant growth due to constitutive gene overexpression. Although several tissue- or organ-specific promoters were used for trait improvement, including green tissue-, embryo-, endosperm-, and root-specific promoters [41,42,43,44,45], the exploration of callus-specific promoters remains relatively limited, with very few promoters of this type being available for transformation tests [37,46]. On the other hand, although drought- inducible promoters have been used to drive CRE-mediated excision of the morphogenic genes [32], this approach makes vector construction more challenging and also complicates the transformation procedure. Therefore, the identification and use of callus-specific promoters may provide an alternative solution to the side effects of the morphogenic genes.

To identify callus-specific promoters, we first screened out 24 rice (*O. sativa* L.) callus-specific genes from transcriptome databases in this study, of which 12 were further selected based on qPCR analysis. The spatiotemporal expression patterns of the promoters of five of these genes were qualitatively analyzed by fusing them with the β-glucuronidase (*GUS*) reporter gene. Finally, the promoter *pOsTDL1B* was chosen, and its callus specificity and expression levels were further verified through the quantification of GUS levels in transgenic rice (*O. sativa* L.). The promoters characterized in this study offer potential alternatives for minimizing the deleterious effects of morphogenic genes in crop transformation.

## 2. Materials and Methods

### 2.1. Plant Materials

*Japonica* rice (*O. sativa* L.) variety Kitaake was used for promoter cloning and plant transformation experiments in this study. After surface sterilization, the rice seeds were cultured for 28 days. The calli were subsequently transferred first to a shoot regeneration medium until the regenerated shoots reached approximately 1 cm and then to a fresh rooting medium for 15 days to induce root formation. Samples were collected from 13-day-old regenerated shoots cultured on the regeneration medium and 15-day-old seedling stems, leaves, and roots growing on rooting medium, frozen in liquid nitrogen, and stored at −80 °C [47].

### 2.2. RNA Isolation and Gene Expression Analysis

A total of 24 genes that were specifically expressed in calli were identified from Gramene (http://www.gramene.org, accessed on 18 February 2025). The expression profiles of these genes are listed in Supplemental Table S1. Total RNA was separately isolated from several fresh rice tissue samples using TRIzol reagent (Thermo Fisher Scientific, Waltham, MA, USA). The first-strand cDNA was generated by employing reverse transcriptase (Takara, Dalian, China). The samples contained subcultured calli grown on subculture medium for 10 days, regenerated calli, regenerated shoots grown on regeneration medium for 15 days, and mixed samples containing roots, stems, and leaves prepared from 13-day-old seedlings grown on rooting medium. qPCR was performed using an Applied Biosystems 7500 Real-Time PCR system and TaKaRa SYBR Green qPCR mix (TaKaRa, Dalian, China). The sequences of the primers used for the qPCR assay are listed in Supplementary Table S2. For each sample, gene expression levels were normalized to those of the ubiquitin (*UBI*) gene and were quantitated using 2^−ΔΔCt^ method [48]. The expression levels in mixed samples containing roots, stems, and leaves from 15-day-old seedlings grown on rooting medium were used as a reference for calculating fold changes in expression. The gene expression data were analyzed using GraphPad Prism 8 software. Error bars indicate standard deviation (SD).

### 2.3. Promoter Cloning, Vector Construction, and Sequence Analysis

DNA sequences spanning approximately 2 kb upstream of the translation start sites of 12 rice callus-specific genes from the Kitaake genome were defined as promoter regions and used for subsequent analysis. These promoters were named P_060_, *pOsTDL1B*, *pOsEDC*, *pOsDLN53*, P_A5P3_, P_B3P2_, P_C1P2_, P_C2P2_, P_D1P4_, P_D2P4_, P_D4P1_, and P_L779_, respectively. The promoter fragments were PCR-amplified from the rice variety Kitaake using promoter-specific primers. The constitutive CaMV 35S promoter served as a positive control. To generate promoter–GUS fusion constructs, after digestion with EcoRⅠ and NcoⅠ, the amplified promoters were inserted into the 1305:*GUS* vector using In-Fusion cloning (Catalog#: 638948; TaKaRa, Dalian, China), replacing the 35S promoter driving *GUS* expression. The constructs were verified by Sanger sequencing. The primers used for the amplification of the different promoter sequences and sequencing are provided in Supplementary Table S2. The *pOsTDL1B* promoter sequence is shown in Supplementary Table S3. *Cis*-acting elements were identified by employing the PlantCARE website (http://bioinformatics.psb.ugent.be/webtools/plantcare/html/).

### 2.4. Generation of Transgenic Rice Plants

The completed constructs were introduced into calli using a modified *Agrobacterium*-mediated transformation method [47]. Dehulked *japonica* rice (*O. sativa* L.) variety Kitaake seeds were surface-sterilized for 20 min in 20% sodium hypochlorite, rinsed with distilled water, and then used to induce callus formation. Calli were induced on the N6 medium supplemented with B5 vitamines, 2,4-D 2 mg/L, proline 2.8 g/L, and sucrose 30 g/L. Four-week-old calli were infected with *Agrobacterium*. After three days of co-culture, the infected calli were cultured in a hygromycin-containing medium. Every 2 weeks, infected calli were subcultured on fresh screening medium until hygromycin-resistant calli developed. At the end of selection, calli were stained with X-Gluc to detect *GUS* expression. These hygromycin-resistant calli were allowed to grow for 2 weeks on the regeneration medium, transferred to rooting medium, and the resulting seedlings were transplanted to the greenhouse and grown to maturity. T_1_ seeds were harvested and used to induce calli. The calli induced from T_1_ seeds were stained again to confirm *GUS* expression.

### 2.5. DNA Extraction and Identification of T_1_ Generation Transgenic Rice Plants

Transgenic rice leaves were collected approximately 20 days after the seeds were sown. The presence of promoter–GUS constructs in the rice genome was confirmed by PCR. Genomic DNA was isolated from the transgenic rice leaves using the cetyltrimethylammonium bromide (CTAB) method [49]. Positive transgenic T_1_ plants were used for further analysis.

### 2.6. Histochemical Staining

Transgenic plants were subjected to GUS staining at various developmental stages and in various tissues, as previously described [50]. Transgenic plant samples were submersed in staining buffer solution containing X-Gluc and incubated at 37 °C for 4 h in darkness. After staining, the samples were submerged in 95% (*v*/*v*) ethanol at room temperature to remove chlorophyll. Stained tissue was imaged with a Leica M305FCA fluorescence stereomicroscope equipped with a DMC6200 camera (Leica Instruments Co., Ltd., Wetzlar, Germany).

### 2.7. Quantification of GUS Activity

To further verify the specificity of the promoter, rice calli were induced from T_1_ transgenic seeds harboring *pOsTDL1B:GUS* in the dark for 15 days at 28 °C. GUS-positive calli from each transgenic event were individually expanded for up to 30 days during the screening stage. Then, calli from each independent transformation event were transferred to regeneration medium and cultured for 13 days, following which the regenerated independent transgenic plantlets were moved onto rooting medium and cultured for 15 days. For each transgenic event, the numbering of the calli corresponded to the numbering of the regenerated shoots grown on the regeneration medium and seedlings grown on rooting medium. Various tissues from different transgenic events at different developmental stages were taken separately, shock-frozen in liquid nitrogen, and stored at −80 °C.

Approximately 100 mg of frozen plant materials, including calli, regenerated shoots, roots, stems, and leaves of seedlings cultured on rooting medium, were homogenized into fine powder in liquid nitrogen. Then, the supernatant was used for quantitative measurements of GUS activity. A 200 μL volume of the resulting protein extract was mixed with 4-methylumbelliferyl-*β*-*D*-glucuronide substrate and incubated at 37 °C for 1 h. The reaction was terminated by transferring 20 μL of the reaction mix to a 96-well plate containing 180 μL of 0.2 M Na_2_CO_3_. The 4-methylumbelliferone (4-MU) fluorescence intensity was determined at emission and excitation wavelengths of 365 and 455 nm, respectively, using a microplate reader [50]. Three independent T_1_ lines were investigated for each construct. Each biological sample was quantified in triplicate. The results are presented as means; error bars indicate standard deviation (SD).

## 3. Results

### 3.1. Identification of Genes Specifically Expressed in Rice Calli

**Figure 1:**
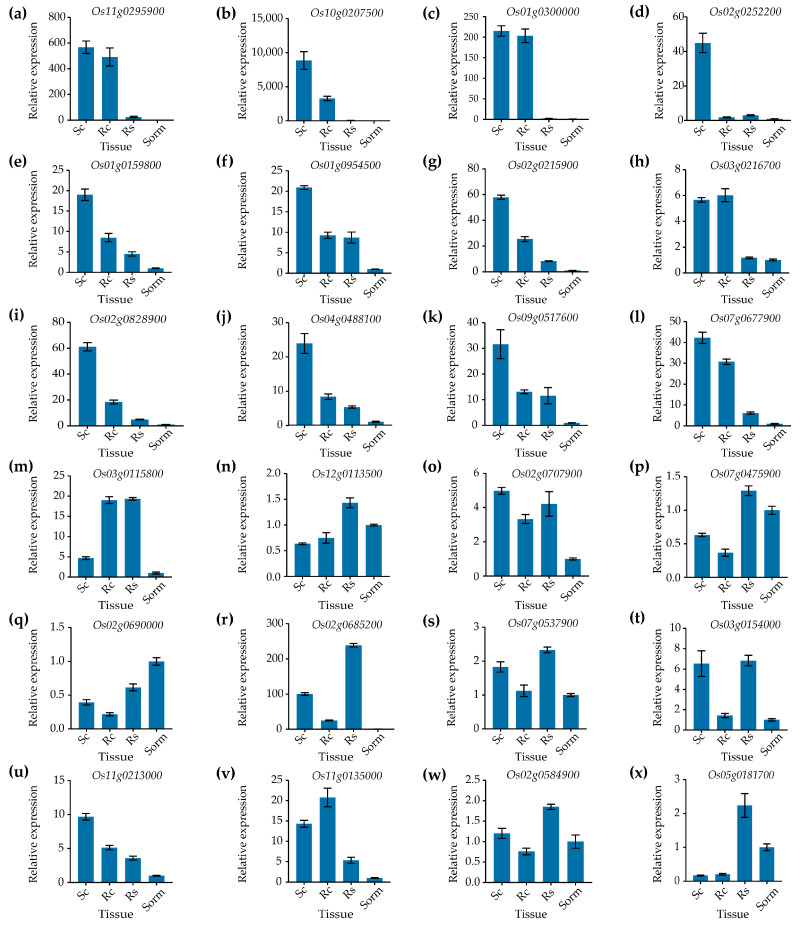
Identification of callus-specific genes by qPCR. (**a**–**l**) The expression levels of the candidate callus-specific genes. (**m**–**x**) The expression levels of the genes with low tissue specificity. Sc, subcultured callus; Rc, regenerated callus; Rs, regenerated shoot; Sorm, the mixed sample containing roots, stems, and leaves of seedlings grown on rooting medium for 15 days. The *UBI* gene served as an internal control. Data are presented as means ± SD (*n* = 3).

To identify genes highly expressed in callus relative to other tissues, qPCR was performed to measure the relative expression levels of the 24 candidate genes in four tissues (callus at 10 days after subculture, regenerated callus cultured on regenerated medium for 13 days, regenerated shoots grown on regenerated medium for 13 days, and seedlings grown in rooting medium for 15 days). Among the 24 genes tested, 12—*Os11g0295900*, *Os10g0207500*, *Os01g0300000*, *Os02g0252200*, *Os01g0159800*, *Os01g0954500*, *Os02g0215900*, *Os03g0216700*, *Os02g0828900*, *Os04g0488100*, *Os09g0517600*, and *Os07g0677900*—displayed callus specificity (Figure 1a–l). The remaining genes showed no, or nearly no, callus-specific expression patterns (Figure 1m–x). The expression levels of the *Os11g0295900*, *Os10g0207500*, *Os01g0300000*, *Os02g0252200*, and *Os04g0488100* genes in subcultured calli of rice were 568.0-, 8859.0-, 215.0-, 44.9-, and 23.8-fold higher, respectively, than those of the mixed sample containing roots, stems, and leaves of 15 days-old seedlings cultivated on rooting medium (Figure 1a–d,i). Moreover, the expression levels of all five genes were very low in regenerated shoots and the mixed sample containing roots, stems, and leaves of 15-day-old seedlings grown on rooting medium. This demonstrated that all five genes exhibited high specificity of expression in the callus.

### 3.2. The Genetic Validation of Promoter Activities

To verify the callus specificity of the candidate promoters, transgenic rice plants harboring the promoter–GUS fusions were produced successfully through *Agrobacterium*-mediated transformation. We detected GUS activity in various tissues at different developmental stages through GUS staining. For each of these vectors (*pOsTDL1B:GUS*, *pOsEDC:GUS*, *pOsDLN53:GUS*, *P_060_:GUS*, *P_D2P4_:GUS*), we obtained at least 20 independent T_0_ generation transgenic lines, and 15, 10, 10, 3, and 3 T_1_ transgenic lines, respectively. The phenotypes of both T_0_ and T_1_ generation transgenic lines did not significantly differ from those of wild-type plants and were used for subsequent analysis.

**Figure 2:**
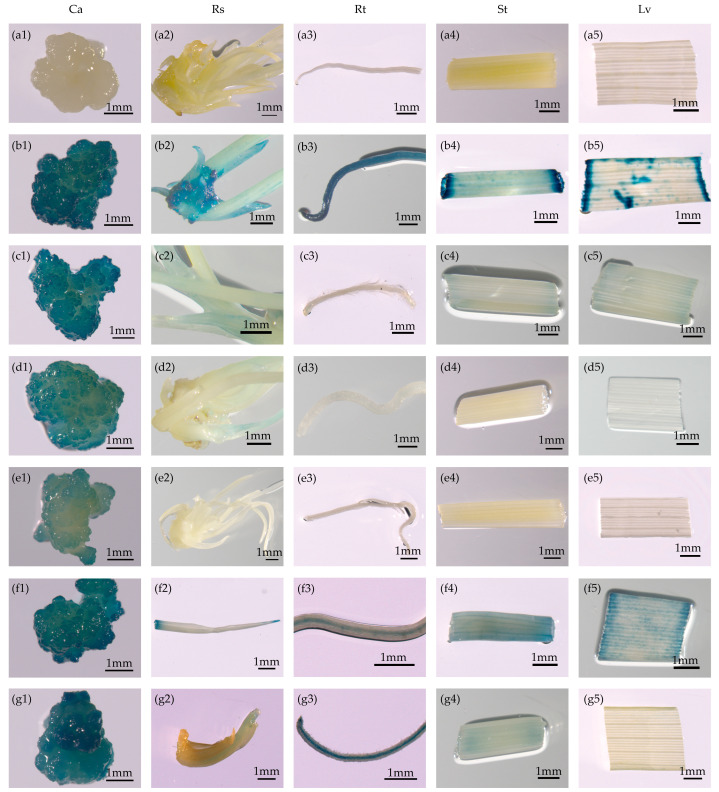
Promoters with callus-preferred expression, as shown by GUS staining. (**a1**–**a5**) Non-transformed control. (**b1**–**g5**) The reporter vectors containing the GUS gene driven by the 35S, *pOsTDL1B*, *pOsEDC*, *pOsDLN53*, P_D2P4_, and P_060_ promoters, respectively. Ca, Rs, Rt, St, and Lv represent calli; regenerated shoots cultured on regeneration medium; and roots, stems, and leaves of seedlings grown on rooting medium of T_0_ transgenic rice, respectively. Scale bars = 1 mm.

Histochemical GUS staining revealed the expression intensity and patterns of the promoters in calli (30 days old), shoots (13 days old) regenerated on regeneration medium, and roots, stems, and leaves of seedlings (15 days old) grown on rooting medium. The different vectors carrying GUS driven by different promoters reflected different activities based on sites of integration and the gene copy numbers in the genome. We detected strong GUS activity in the transgenic rice calli carrying *pOsTDL1B:GUS* and *P_D2P4_:GUS* (Figure 2c1–c5,f1–f5). We also observed moderately intense GUS activity in calli harboring the GUS reporter gene under the control of the *pOsEDC* and P_060_ promoters (Figure 2d1–d5,g1–g5). GUS expression in calli was relatively weak under the *pOsDLN53* promoter compared with that under the other four promoters (Figure 2e1–e5).

For further analysis of the tissue and temporal specificity of the putative callus-specific promoters, GUS staining was detected in 13-day-old shoots regenerated on regeneration medium and roots, stems, and leaves of 15-day-old seedlings grown on rooting medium (Figure 2). GUS activity under the control of the *pOsTDL1B* promoter was relatively weak among the four non-callus tissues tested (Figure 2c1–c5), suggesting that the expression of *pOsTDL1B:GUS* was primarily restricted to callus. Compared with callus tissue, the *pOsEDC* promoter drove no or only slight GUS expression in the other tissues examined (Figure 2d1–d5), indicating that this promoter was also callus-specific. For the *pOsDLN53:GUS* construct, GUS activity was barely visible among the above-mentioned non-callus tissues tested (Figure 2e1–e5), indicating that GUS expression driven by this promoter was strictly restricted to callus. GUS expression driven by the P_060_ promoter showed only medium intensity in callus but was stronger in roots grown on rooting medium than in calli (Figure 2g1–g5). This suggested that the P_060_ promoter may serve as a potential root-specific promoter. For the P_D2P4_:GUS transgenic line, strong GUS activity was observed in the various tissues analyzed, including regenerated shoots cultured on regeneration medium and the roots, stems, and leaves of seedlings grown on rooting medium (Figure 2f1–f5). These results indicated that three out of the five promoters (*pOsTDL1B*, *pOsEDC*, and *pOsDLN53*) drove only relatively low expression in non-callus tissues.

**Figure 3:**
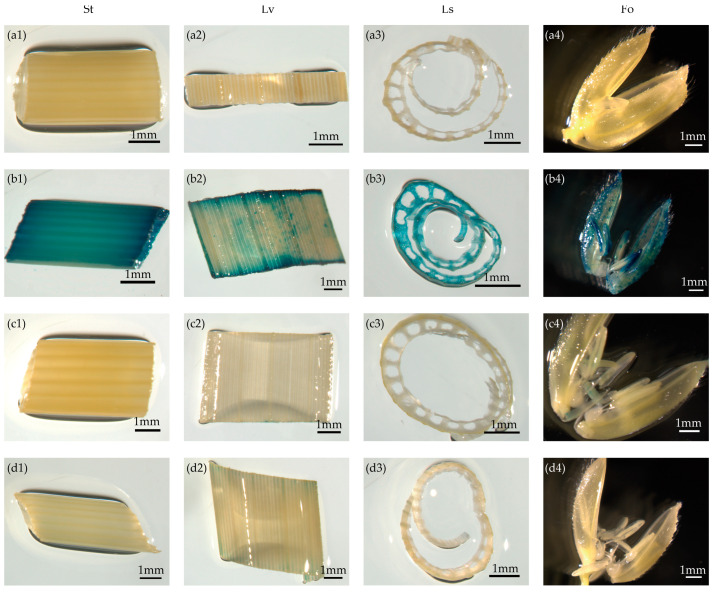
Non-detectable expression of callus-specific promoters in vegetative tissues rather than callus as shown by GUS staining. (**a1**–**a4**) Non-transformed control. (**b1**–**d4**) Reporter vectors containing the GUS gene driven by the 35S, *pOsTDL1B*, and *pOsEDC* promoters, respectively. St, Lv, Ls, and Fo represent stems, leaves, leaf sheaths, and floral organs, respectively, of T_1_ generation transgenic rice plants at the heading stage. Scale bars = 1 mm.

To further verify the expression patterns of the candidate promoters, we investigated GUS activity in stems, leaves, leaf sheaths, and floral organs at the heading stage in T_1_ *pOsTDL1B*:*GUS* and *pOsEDC*:*GUS* plants grown under natural conditions. GUS staining in *pOsTDL1B:GUS* transgenic rice was weak or barely detectable in the tested tissues and organs (Figure 3c1–c4). GUS expression driven by the *pOsEDC* promoter was very low in all tested tissues at the heading stage (Figure 3d1–d4). This further indicated that the activities of these two promoters in the four different tissues were very low or almost undetectable at this stage.

Taken together, the above results confirmed that the five cloned promoters can direct GUS expression across the nine tested tissue types, but only three—*pOsTDL1B*, *pOsEDC*, and *pOsDLN53*—showed high callus specificity, with the *pOsTDL1B* promoter exhibiting the most significant effects. The *pOsEDC* promoter preferentially drove GUS expression in calli and exhibited medium intensity. Although the strength of the *pOsDLN53* promoter in rice callus was not as intensive as that of *pOsTDL1B* or *pOsEDC*, it nonetheless displayed more stringent callus specificity. However, P_D2P4_ and P_060_ promoter-driven expression was not restricted to callus.

### 3.3. Quantitative GUS Activity in pOsTDL1B:GUS Transgenic Rice

**Figure 4:**
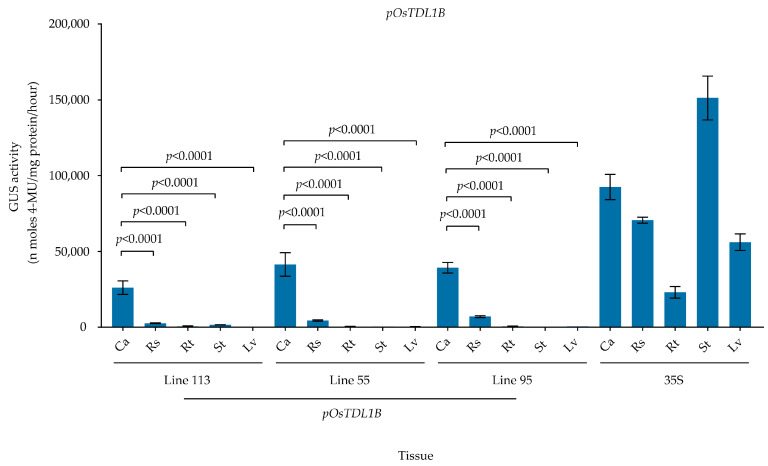
Quantification of GUS activity in transgenic rice carrying the *pOsTDL1B:GUS* construct. Calli, regenerated shoots cultured on shoot-regeneration medium, and roots, stems, and leaves of seedlings grown on rooting medium were used for extraction of total proteins. Lines 113, 55, and 95 are three independent transgenic events for the *pOsTDL1B* vector. Ca, Rs, Rt, St, and Lv represent calli, regenerated shoots cultured on regeneration medium, and roots, stems, and leaves of T1 seedlings grown on rooting medium. 35S is a constitutive CaMV 35S promoter (35S:GUS) that served as a positive control. Data are shown as means ± SD (*n* = 3). *p*-values were determined using one-way ANOVA test with Tukey’s multiple comparisons test. Ns represents no significance.

To further define the callus specificity of the *pOsTDL1B* promoter, GUS activity was quantitated during rice tissue culture. For this, a total of 15 independent transgenic rice lines containing the *pOsTDL1B:GUS* construct were generated. We selected three of these lines (113, 55, and 95) with different GUS expression intensities in callus for further analysis of GUS activities. The strength of the *pOsTDL1B* promoter was assessed by measuring GUS expression in 30-day-old calli from the three independent transgenic lines. In the T_1_ generation, GUS activity varied greatly among the three lines. Mean GUS activity in calli from the three independent transgenic lines (*pOsTDL1B*#113, *pOsTDL1B*#55, and *pOsTDL1B*#95) was 26,088.1, 41,466.9, and 39,249.0 nmol 4 MU/mg protein/hour, respectively (Figure 4). Furthermore, for *pOsTDL1B*#113, GUS activity in calli cultured for 30 days, regenerated shoots cultured on regeneration medium for 13 days, and roots and stems of 15-day-old seedlings grown on rooting medium was 317.8, 32.1, 6.8, and 19.8 times that of leaves cultured on rooting medium for 15 days. Regarding *pOsTDL1B*#55, GUS activity in calli cultured for 30 days, regenerated shoots cultured on regeneration medium for 13 days, and roots and stems of 15-day-old seedlings grown on rooting medium was 106.0, 11.2, 1.0, and 1.2 times that of the leaves of 15-day-old seedlings cultured on rooting medium. As for *pOsTDL1B*#95, GUS activity in calli cultured for 30 days, regenerated shoots cultured on regeneration medium for 13 days, and roots and stems of 15-day-old seedlings grown on rooting medium was 63.9, 11.4, 1.1, and 0.6 times that of the leaves of 15-day-old seedlings cultured on rooting medium.

Together, these results indicate that among the three transgenic lines, calli harboring *pOsTDL1B:GUS* exhibited markedly higher GUS activity than regenerated shoots grown on regeneration medium and roots, stems, and leaves of seedlings grown on rooting medium. Three independent transgenic lines exhibited consistent *GUS* expression patterns with high callus specificity (Figure 4). This was mostly consistent with the *Os10g0207500* expression pattern observed through qPCR analysis and *pOsTDL1B* promoter activity confirmed by GUS staining in transgenic rice lines, suggesting that the *pOsTDL1B* promoter exhibits high callus specificity.

### 3.4. Analysis of Cis-Acting Elements in the pOsTDL1B Promoter

**Figure 5:**
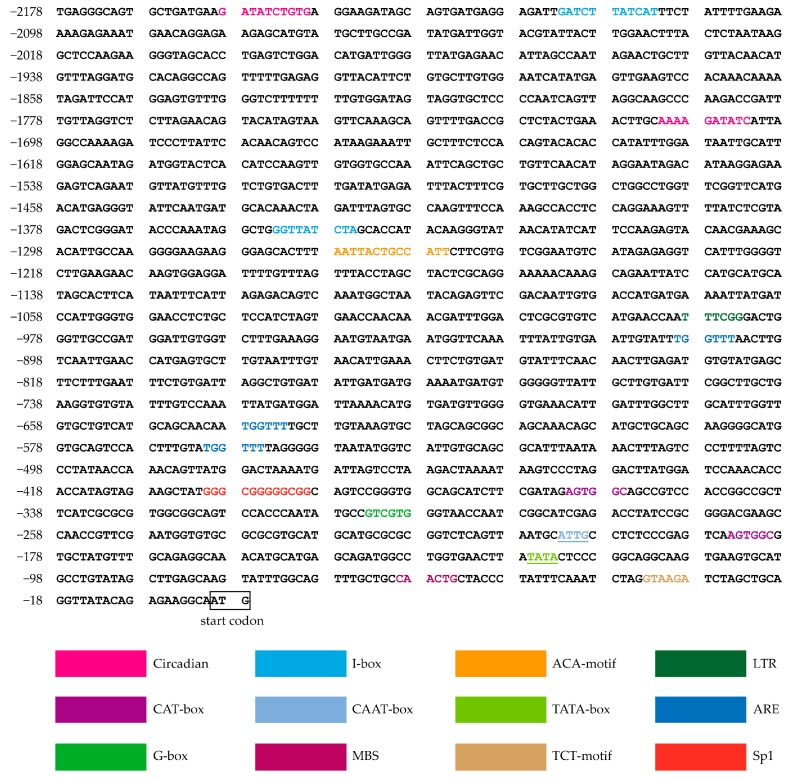
Analysis of *cis*-acting elements in the *pOsTDL1B* promoter based on the PlantCARE database. CAT-box indicates a *cis*-acting regulatory element associated with meristem expression; TCT-motif, I-box, Sp1, G-box, and ACA-motif indicate *cis*-acting regulatory elements involved in light responsiveness; MBS denotes a MYB binding site associated with drought-inducibility; LTR refers to low-temperature response elements; TATA-box and CAAT-box are core promoter elements; ARE denotes an anaerobic response element.

To assess the presence of *cis*-acting elements, promoters were predicted for the 2178 bp upstream of the ATG translation start site. The *cis*-acting elements identified in the *pOsTDL1B* promoter are shown in Figure 5. In addition to a putative TATA box and a CAAT box, several other responsive elements were found in the *pOsTDL1B* promoter. The *cis*-acting regulatory elements detected included seven light-responsive elements (TCT-motif, Sp1, I-box, G-box, ACA-motif), suggesting that the promoters may be involved in callus formation and callus-specific gene expression through the regulation of light. The identified elements also included three *cis*-acting regulatory elements critical for the anaerobic induction at different locations (anaerobic responsive elements, ARE), two elements associated with circadian rhythm control (circadian), a low temperature-responsive (LTR) element box, two *cis*-acting elements associated with meristem expression (CAT-box), and a drought response element (MYB binding site, MBS).

## 4. Discussion

In this research, we report that we screened out 24 callus-specific genes employing a public database, 12 of which were identified specifically in the expression of callus through qPCR. GUS staining showed that the *pOsTDL1B* promoter exhibits strong callus-specific expression. *pOsEDC* is specifically expressed in callus and has a medium strength. *pOsDLN53* is a weak promoter with relatively strict expression activity. Quantitative analysis of GUS activity confirmed that all three *pOsTDL1B:GUS* independent transgenic lines tested in this study display high specificity of expression in the callus. These results were consistent with those obtained using qPCR and GUS staining analyses, implying that the *pOsTDL1B* promoter is strongly callus-specific. Accordingly, the callus-specific promoter identified in this study holds promise for avoiding deleterious effects on the host due to continuous high-level expression of the morphogenic genes in all tissues [32,33].

Promoter-driven specific expression plays a crucial role in gene editing and breeding programs. Some *cis*-elements conferring tissue specificity have been identified. It has been shown that the GCN4 motifs are critical for endosperm specificity utilizing a stable transgenic system [51]. A TCCAAAA motif was found to confer fruit-specific expression in the watermelon (*Citrullus vulgaris* S.) *AGPL1* promoter [52]. Several *cis*-regulatory elements, including LPSE1, LPSRE1, and LPSE2, have been characterized in the green tissue-specific promoter *P_D54O_* [53]. In addition, in rice, GSE1 and GSE2 were also identified as *cis*-acting elements that control green tissue-specificity in the *P_DX1_* promoter [54]. Here, *cis*-acting elements in the callus-specific promoter *pOsTDL1B* were investigated using PlantCARE. Surprisingly, we found that this promoter did not contain the motif that is directly involved in callus-specific expression. This phenomenon is similar to that reported for the endosperm-specific *GluC* promoter and non-endosperm tissue-expressed promoters P_OsNETE1_-P_OsNETE5_ [55,56]. However, many LREs, such as TCT-motif, Sp1, I-box, G-box, and ACA-motif, were found in the promoter *pOsTDL1B*. Because callus formation occurs in the absence of light, we speculate that these LREs may directly and/or indirectly affect callus formation and callus-specific gene expression. Therefore, a systematic investigation is required to determine whether the LREs in the *pOsTDL1B* promoter contribute to the regulation of these processes.

This study focused on the identification of promoters that are specifically and highly expressed in calli. However, some promoters showing weak or moderately strong activity were also identified by qPCR and GUS staining. Moderately strong promoters can drive high levels of protein expression [57]. In a previous study, the weak promoter *Axig1* was used to drive the tissue-specific expression of *Wus2* genes [58]. Similarly, the moderately strong promoter P_060_ and the weak promoter *pOsEDC* identified in this study may also be exploited to enable plants to express foreign proteins, specifically in callus tissue. Medium and weak promoters provide flexible and ideal candidates for rice transgenic engineering and multigene-stacking approaches in crops. The weak *pOsDLN53* promoter identified in this study showed strict callus-specificity. In addition to direct use, this promoter can be modified by adding auxiliary components to increase its strength and further expand its scope and utility. Furthermore, we found that the callus-specific promoter P_060_, in addition to being moderately expressed in calli, was also highly expressed in roots. The utilization of root-specific promoters may help increase tolerance to both biotic (e.g., pests, pathogens, and malnutrition) and abiotic (e.g., drought) stresses. Accordingly, the P_060_ promoter may serve as a useful research tool for crop breeding and engineering.

Morphogenic genes are receiving increasing attention because transformation frequencies of recalcitrant genotypes can be significantly enhanced by the overexpression of these genes [59,60,61]. However, the constitutive expression of *Bbm* and *Wus2* often confer severe pleiotropic defects [32,33]. There are two main approaches to regulate morphogenic genes: a chemical-inducible system and a heat shock-inducible FRT/FLP system [35,62]. Utilizing the genetic transformation systems that rely on inducible excision or chemical induction to control morphogenic gene expression can stimulate plant growth and avoid negative impacts on plant growth and fertility. To date, only a limited number of callus-specific promoters have been employed in rice genetic transformation [46]. Drought-inducible promoter *rab17* was previously utilized to offer the control of CRE-mediated excision to overcome pleiotropic effects caused by morphogenic regulators [32]. However, this adds complexity to vector construction and the transformation procedure, with the added risk that the excised morphogenic genes may reintegrate into the genome [32,63]. A maize phospholipid transferase protein (Zm-PLTP) promoter was identified as being non-constitutive and was used to drive the expression of the morphogenic genes *Bbm* and *Wus*, thereby mitigating potential negative effects [58]. This report further supports the feasibility and application potential of our proposed approach, which involves utilizing tissue-specific or stage-specific promoters to direct the expression of genes, thereby avoiding the adverse effects associated with constitutive promoters.

Although significant progress has been made over the past decades in rice genetic transformation. Currently, genotype dependency, which is the major influential factor in rice genetic transformation, restricts the ability of rice improvement through transgene integration and genome-editing methods. Constitutional expression of morphogenic genes can significantly help enhance plant transformation efficiency. However, they can also lead to adverse effects, such as phenotypic abnormalities and sterility. Herein, we have identified and characterized a callus-specific promoter, which can be used to restrict the spatiotemporal expression of morphogenic genes (*Bbm* and *Wus*); therefore, it may help to mitigate the negative effects of morphogenic genes. The application of the promoter may improve the regeneration efficiency of some recalcitrant indica and *japonica* varieties. Additionally, it can be used for other species such as sorghum (*S. bicolor* L.) and maize (*Z. mays* L.) to improve transformation efficiency and thereby enhance gene-editing efficiency, reduce the cost of genetic transformation, and accelerate the breeding process.

## 5. Conclusions

We screened 24 callus-specific genes utilizing public databases, and 12 of 24 genes tested by qPCR were validated to be callus specific. Five promoters out of twelve genes were cloned. Three out of five promoters have been shown to display callus-specific properties and have different strengths confirmed by GUS staining. The quantification of GUS activity further confirmed that *pOsTDL1B* promoter is a strong callus-specific promoter. Our research offers a callus-specific promoter, which is suitable for decreasing the negative impact resulting from constitutive overexpression of morphogenic regulatory genes.

## Data Availability

The original contributions presented in this study are included in the article/Supplementary Materials. Further inquiries can be directed to the corresponding author.

## Supplementary Materials

The following supporting information can be downloaded at https://www.mdpi.com/article/10.3390/genes16050610/s1, Table S1: The expression patterns of callus-preferential genes screened from the Gramene database; Table S2: Primer sequences; Table S3: The sequence of *pOsTDL1B*.

## Abbreviations

qPCR	quantitative real-time polymerase chain reaction
MTF	morphogenic transcription factor
CTAB	cetyltrimethylammonium bromide
4-MU	4-methylumbelliferone
